# A Brief Review of Research Using Near-Infrared Spectroscopy to Measure Activation of the Prefrontal Cortex during Emotional Processing: The Importance of Experimental Design

**DOI:** 10.3389/fnhum.2016.00529

**Published:** 2016-10-20

**Authors:** Robert C. A. Bendall, Peter Eachus, Catherine Thompson

**Affiliations:** Directorate of Psychology and Public Health, School of Health Sciences, University of SalfordSalford, UK

**Keywords:** emotion, prefrontal cortex, near-infrared spectroscopy, review, mood, neuroimaging, affective neuroscience

## Abstract

During the past two decades there has been a pronounced increase in the number of published research studies that have employed near-infrared spectroscopy (NIRS) to measure neural activation. The technique is now an accepted neuroimaging tool adopted by cognitive neuroscientists to investigate a number of fields, one of which is the study of emotional processing. Crucially, one brain region that is important to the processing of emotional information is the prefrontal cortex (PFC) and NIRS is ideally suited to measuring activity in this region. Compared to other methods used to record neural activation, NIRS reduces the discomfort to participants, makes data collection from larger sample sizes more achievable, and allows measurement of activation during tasks involving physical movement. However, the use of NIRS to investigate the links between emotion and cognition has revealed mixed findings. For instance, whilst some studies report increased PFC activity associated with the processing of negative information, others show increased activity in relation to positive information. Research shows differences in PFC activity between different cognitive tasks, yet findings also vary within similar tasks. This work reviews a selection of recent studies that have adopted NIRS to study PFC activity during emotional processing in both healthy individuals and patient populations. It highlights the key differences between research findings and argues that variations in experimental design could be a contributing factor to the mixed results. Guidance is provided for future work in this area in order to improve consistency within this growing field.

## Introduction

Near-infrared spectroscopy (NIRS) is a non-invasive neuroimaging technique that measures changes in concentrations of specific chromophores (oxygenated hemoglobin [oxy-Hb] and deoxygenated hemoglobin [deoxy-Hb]) in biological tissue. Like functional magnetic resonance imaging (fMRI), NIRS relies on the principle of neurovascular coupling (the relationship between cerebral blood flow and neural activity) to infer brain activity from changes in oxygenation. The first NIRS studies were published in 1993 (e.g., Chance et al., 1993) and during the past two decades there has been a dramatic rise in the use of NIRS as a neuroimaging technique in cognitive neuroscience research. Whilst NIRS does have limitations in comparison to other neuroimaging techniques, including its poor temporal resolution compared to electroencephalography (EEG) and poor spatial resolution compared to fMRI, it also offers numerous advantages. NIRS is easily combined with other neuroimaging techniques including EEG (Herrmann et al., 2008) and has a higher temporal resolution than fMRI. Additionally, NIRS can reduce the discomfort to participants, make data collection from larger sample sizes more feasible, and individuals with non-removable metal objects in their body or who have tattoos are not excluded. Further, NIRS allows for the measurement of brain activation during a variety of tasks (e.g., during exercise; Lucas et al., 2012). Lastly, due to the fact that it is less invasive than other techniques, NIRS permits the monitoring of brain activation from certain populations that would otherwise be more difficult to access, such as infants (Franceschini et al., 2007), the elderly (Mehagnoul-Schipper et al., 2002), and patient populations (Matsuo et al., 2004; Matsubara et al., 2014; Yokoyama et al., 2015; Ruocco et al., 2016).

Crucial to the current work, NIRS is a useful technique for studying the influence of emotion. It is proposed that during an emotional situation, or when presented with emotional information, two competing processing strategies are present. Emotion influences cognition in a bottom-up manner regardless of the demands of a task, whilst simultaneously individuals adopt top-down cognitive control strategies to direct resources to task completion and emotion regulation. Emotional control therefore involves higher order emotion-related regions, including the prefrontal cortex (PFC), and it is hypothesized that the degree of success in the recruitment of prefrontal neural systems has a direct impact on task performance in emotional situations. As NIRS can only measure neural activation from cortical structures approximately 1–2 cm deep in the brain, it cannot record amygdala-generated neural activation from the limbic system, an area traditionally associated with emotional processing. However, NIRS is ideal for recording PFC neural activation and has been adopted by cognitive neuroscientists investigating the influence of emotion on cognition (Doi et al., 2013).

Having outlined the suitability of NIRS as a tool for measuring emotion-related neural activation, the next stage is to review evidence from NIRS research implicating the PFC in tasks incorporating emotional processing. Research will be reviewed from studies measuring PFC activation during the presentation of emotional stimuli (Table 1), studies recording activity during task completion where naturally occurring emotion is recorded or emotion is artificially induced (Table 1), and from studies of PFC neural activation in patient populations (Table 2). Differences in experimental findings will be discussed in relation to the chosen experimental design. There are studies using NIRS to investigate emotional processing in brain regions outside of the PFC (e.g., Köchel et al., 2011; Plichta et al., 2011) and whilst processing emotional faces (e.g., Sun et al., 2015; Rodrigo et al., 2016), however these are outside the scope of the current review. A number of recent articles have also covered methodological issues including filtering of NIRS data and artifact rejection (Guerrero-Mosquera et al., 2016; Kamran et al., 2016; Tachtsidis and Scholkmann, 2016) and these are also not covered in the present work. It should also be noted that in this review we focus on experimental findings that report concentration changes in oxy-Hb. NIRS measures changes in oxy-Hb, deoxy-Hb, and compound activity (oxy; oxy-Hb–deoxy-Hb) and it is been argued that changes in oxy-Hb concentrations are the most reliable measure of cerebral blood flow and, by extension, neural activity (Malonek et al., 1997; Strangman et al., 2002). Despite this, it has been suggested that deoxy-Hb has a closer relation to the blood oxygen level dependent response in fMRI, and simultaneously reporting both oxy-Hb and deoxy-Hb allows better physiological interpretations to be made (Tachtsidis and Scholkmann, 2016).

**Table 1:** **Key findings from studies measuring PFC activation during passive viewing of emotional stimuli and cognitive tasks**.

**References**	**Participants**	**Method**	**Analysis**	**Main findings**
Glotzbach et al., 2011	Healthy adults	Passive viewing of neutral and negative (fearful) images	Group analysis of oxy-Hb model-based contrasts between task conditions	↑ oxy-Hb for negative stimuli compared to neutral stimuli in 4 channels located above the precentral lobe, inferior frontal cortex, superior medial cortex, and orbital frontal cortex
Hoshi et al., 2011	Healthy adults	Passive viewing of positive and negative images	Individual and group analysis of oxy-Hb during task conditions compared to baseline oxy-Hb	Individual analysis ↑ oxy-Hb for 1 participant and ↓ oxy-Hb in 9 participants for positive stimuli. ↑ oxy-Hb for 7 participants and ↓ oxy-Hb for 6 participants for negative stimuli. Group analysis ↑ oxy-Hb in 2 channels (right and left vlPFC) for negative stimuli. ↓ oxy-Hb activity in 1 channel (adjacent to left dlPFC) for positive stimuli. All compared to baseline
Herrmann et al., 2003	Healthy adults	Passive viewing of positive, neutral, and negative images	Group analysis of oxy-Hb during task conditions and baseline oxy-Hb	No differences in oxy-Hb when viewing positive, neutral, and negative images compared to baseline
Ozawa et al., 2014	Healthy adults	Passive viewing of neutral and negative stimuli	Individual and group analysis of oxy-Hb during stimuli presentation compared to baseline oxy-Hb. Group analysis of oxy-Hb also compared between task conditions	Individual analysis ↑ oxy-Hb in 7 participants and ↓ oxy-Hb in 3 participants for neutral stimuli. ↑ oxy-Hb in 9 participants and ↓ in oxy-Hb in 6 participants for negative stimuli. All compared to baseline. Group analysis ↑ oxy-Hb for both neutral stimuli (12 channels) and negative stimuli (3 channels) including left and right superior frontal gyrus compared to baseline. No differences between stimuli groups
Aoki et al., 2011	Healthy adults	Verbal WM task	Correlated naturalistic negative mood with oxy-Hb during WM task	↑ levels of naturalistic negative mood correlated with ↓ oxy-Hb during WM task in left dlPFC
Kreplin and Fairclough, 2013	Healthy adults	Image judgment task	Group analysis of oxy compared between task conditions	↑ oxy for positive stimuli compared to negative stimuli in 3 channels located in medial rostral PFC
Ozawa et al., 2014	Healthy adults	n-back WM task	Individual analysis of oxy-Hb during n-back WM task compared to baseline. Group analysis of oxy-Hb compared directly against each other	Individual analysis ↑ oxy-Hb during negative 1-back task in 11 participants (one participant showed a ↓). ↑ oxy-Hb during negative 3-back task in 11 participants (2 participants showed a ↓). Group analysis ↑ oxy-Hb during n-back task after negative stimuli presentation compared to neutral stimuli
Tupak et al., 2014	Healthy adults	Labeling and matching tasks	Group analysis of oxy-Hb contrasts between conditions (task and valence)	Labeling threat vs. control and labeling threat vs. matching threat contrasts revealed ↑ oxy-Hb in both vlPFC and dlPFC. Task x valence interaction ↑ oxy-Hb during labeling but not matching for threat compared to neutral valence. Labeling threatening stimuli ↑ oxy-Hb compared to matching threatening stimuli

**Table 2:** **Key findings from studies measuring PFC activation in patient populations**.

**References**	**Participants**	**Method**	**Analysis**	**Main findings**
Liu et al., 2014	Major depression disorder (MDD) patients and healthy adults	Verbal fluency task	Group analysis of MDD patients and healthy adults. ± in oxy-Hb correlated with depression and anxiety scores	↑ oxy-Hb during verbal fluency task in 37 channels for healthy adults but only 6 channels in MDD patients. ↓ oxy-Hb during verbal fluency task for MDD patients compared to healthy adults in 15 channels. Oxy-Hb positively correlated with depression scores in 8 channels for MDD patients in frontopolar PFC and right dlPFC regions
Matsubara et al., 2014	Bipolar disorder (BD) patients, MDD patients and healthy adults	Emotional stroop task	Group analysis of BD, MDD and healthy adults between task conditions	↑ oxy-Hb during threat task for patients compared to healthy adults. BD patients ↑ oxy-Hb in left inferior frontal region, MDD patients ↑ oxy-Hb in left middle frontal region. For happy words BD patients ↓ oxy-Hb in middle and frontal regions, MDD patients no change
Yokoyama et al., 2015	Social anxiety disorder (SAD) patients and healthy adults	Verbal fluency task	Group analysis of SAD patients and healthy controls during verbal fluency task. ± in oxy-Hb correlated with social anxiety scores	↓ oxy-Hb change in SAD patients vlPFC compared to healthy adults. Right vlPFC oxy-Hb negatively correlated with social anxiety in patients. Healthy adults oxy-Hb positively correlated with social anxiety in vlPFC

## PFC activation during passive viewing of emotional stimuli

The neuroscientific study of emotional processing often involves individuals viewing images of differing emotional valence. This allows the underlying neural networks involved in emotional processing to be investigated. Researchers can create their own emotional stimuli or make use of validated stimuli databases developed by other investigators (e.g., International Affective Picture System; IAPS; Lang et al., 2008; Nencki Affective Picture System; Marchewka et al., 2014). Glotzbach et al. (2011) adopted such an approach using images from the IAPS. The researchers found that bilateral PFC oxy-Hb increased for negative compared to neutral images, suggesting that PFC regions are recruited during processing of negative stimuli. However, these findings are limited because a positive condition was not included. Hoshi et al. (2011) recorded PFC oxy-Hb changes during the presentation of both positive and negative stimuli. This study also found a significant increase in oxy-Hb for negative stimuli but also showed a significant decrease in oxy-Hb for positive stimuli (compared to neutral). Yet, Herrmann et al. (2003) found no differences in oxy-Hb between baseline activity and the viewing of neutral, negative, and positive images. This small collection of studies shows the importance of the stimuli conditions included in experiments investigating emotional processing. Choosing to omit a neutral, negative, or positive stimuli condition means that researchers are not always making the same comparisons within their studies (i.e., whilst some compare negative [and positive] to neutral, others compare negative to positive). This affects the results and makes comparison between studies problematic.

An additional factor that makes it difficult to compare findings is that researchers make use of different analytic approaches. Critically, this seems to have an impact on the findings. Ozawa et al. (2014) analyzed their data by comparing neutral and negative conditions to baseline oxy-Hb and the results suggested increases in oxy-Hb for both types of stimuli. However, when they compared neutral stimuli directly to negative stimuli no differences in oxy-Hb were evident. It may therefore be argued that a comparison to baseline can also reflect task-related activation in addition to any activation due to emotion. Herrmann et al. (2003) compared oxy-Hb in positive, negative, and neutral stimuli to baseline and were able to conclude no influence of emotion in addition to no influence of task. Researchers should ensure baseline data is included in analysis, in combination with control and experimental conditions to allow separation of task-related and emotion-related activation.

A further problem is that whilst some researchers report only group level analysis of PFC activation, others analyze data at both a group and an individual level. When analyzing activation at a group level, Hoshi et al. (2011) found decreased activation in response to positive stimuli and increased activation for negative stimuli. Individual level analysis revealed mostly decreases in oxy-Hb for pleasant stimuli, but both increases and decreases in oxy-Hb for unpleasant stimuli (compared to baseline oxy-Hb). This implies individual variation in the effect of emotional stimuli, but this is not always acknowledged. The choice of analysis clearly has important implications for the interpretation of results.

## PFC activation during task completion

Researchers have also used NIRS to measure activation within more demanding tasks. In this section two types of studies are discussed. First, those investigating the influence of induced or naturally occurring mood on cognitive task performance, and, secondly, studies where the content of the task itself is emotional. Aoki et al. (2011) demonstrated that naturalistic levels of negative mood were negatively correlated with PFC activity during a verbal working memory (WM) task. This is an important observation as it suggests that naturally occurring negative mood influences PFC activity. However, Ozawa et al. (2014) also used a WM task and found *increased* PFC oxy-Hb when participants were induced into negative mood (compared to neutral). Whilst there are differences in the exact nature of the WM tasks used in these two studies [Ozawa et al. (2014) used an n-back task whilst Aoki et al. (2011) adopted a verbal matching task], as well as differences in study design (i.e., using naturally occurring mood vs. induced mood), these conflicting findings show that the relationship between negative mood and cognitive task-related PFC oxy-Hb needs clarifying and warrants further investigation.

Consideration of the associated behavioral data does not necessarily help to explain the differences in these findings. This is particularly the case due to the different practices used by the researchers. For instance, Ozawa et al. (2014) did not analyze task performance in conjunction with oxy-Hb, yet Ogawa et al. (2014) did and found a correlation between increased PFC oxy-Hb and improved performance in a WM task. On the basis of the findings reported by Aoki et al. (2011) it may be predicted that negative mood would lead to poor performance and the researchers did find a relationship between lower negative mood scores and improved verbal WM performance. However, this relationship was no longer significant after controlling for age and gender suggesting that age and gender influence PFC activity during emotional WM tasks, and, that the relationship between mood scores and WM performance is weak. Moreover, Aoki et al. (2011) only observed a weak trend when they correlated PFC oxy-Hb and WM performance. Consequently it is impossible to conclude the association between PFC activation, mood, and task performance.

Adopting a different approach, Tupak et al. (2014) used emotional images embedded in a top-down labeling task and a perceptual matching task to investigate emotional processing. No differences were observed in ventrolateral prefrontal cortex (vlPFC) activity during the labeling task between negative (threatening) and neutral visual stimuli. However, increased vlPFC activity occurred during the matching of threatening images compared to neutral images, showing that the vlPFC is involved during the cognitive evaluation of threatening stimuli.

Kreplin and Fairclough (2013) also incorporated emotion into an experimental task. When participants were asked to make judgments about artistic images they found increased activation for positive stimuli compared to negative stimuli. These findings contrast with studies investigating the passive viewing of emotional images and studies measuring activation during cognitive tasks. However, increases in dorsolateral prefrontal cortex (dlPFC) activity in response to positive stimuli (compared to negative) has also been found using fMRI (Herrington et al., 2005).

One aspect that contributes to the mixed findings is the choice of behavioral task used to measure the effects of emotion on performance. Many researchers make use of WM tasks and this does aid comparison between the different studies, however it also limits the applicability of the findings. An issue arises however when a different task is used. For instance, Bendall and Thompson (2016) induced participants into positive, negative, and neutral moods and measured PFC oxy-Hb in a change detection task and found no difference in activation across emotion conditions. One argument for the non-significant findings was that task difficulty limited the influence of emotion, a claim also supported by behavioral findings (Bendall and Thompson, 2015), as well as the findings of Tupak et al. (2014). Despite this, it can be concluded that it is important to justify the choice of task and to consider the difficulty of the task used in this area of research. Researchers must ensure that the experimental task is selected with some acknowledgment of how the work can compare to other findings in the field.

## PFC activation in patient populations

In addition to measuring the effect of emotion on cognition in general, research has also explored the way in which emotion influences cognitive performance in tasks incorporating emotional information. Altered emotional processing is a key feature in the pathophysiology of mood disorders (Matsubara et al., 2014). For example, it has been shown that increased dlPFC activity when regulating negative affect is correlated with decreases in depression severity (Heller et al., 2013). Furthermore, it has been argued that difficulty initiating top-down control processes required to regulate emotion is a contributing factor in various patient groups or disorders, including major depressive disorder (MDD; Johnstone et al., 2007; Matsubara et al., 2014), bipolar disorder (BD; Matsubara et al., 2014), and borderline personality disorder (New et al., 2008; Krause-Utz et al., 2012).

Matsubara et al. (2014) used an emotional adaptation of the Stroop paradigm (Stroop, 1935) to investigate prefrontal activation in response to emotional words in patients with BD and MDD. They found different patterns of PFC activity for BD and MDD patients compared to healthy controls. In response to threat-related words, BD patients demonstrated increased oxy-Hb in the left inferior frontal region whilst MDD patients showed increased oxy-Hb in the left middle frontal region. Additionally, when responding to happy words, BD patients showed decreased oxy-Hb in the middle frontal regions in both hemispheres, whereas MDD patients showed no significant differences to healthy controls. Moreover, BD patients showed both increased and decreased oxy-Hb in the superior frontal and middle frontal regions compared to MDD patients when responding to happy words. These results suggest that altered PFC neural responses to emotional stimuli may be a trait marker in patient populations (Matsubara et al., 2014). However, additional research is needed to confirm these initial findings. Further, differences in emotion-related oxy-Hb between patient groups suggests that different neural pathways play a role in emotional processing in these disorders.

BD and MDD have been shown to be comorbid with social anxiety disorder (SAD; Kessler et al., 2005) and all three disorders appear to show altered neural responses during emotional processing. Using a verbal fluency task that induced fear of evaluation by others, Yokoyama et al. (2015) measured oxy-Hb and found that SAD patients exhibited smaller increases in vlPFC oxy-Hb compared to healthy controls. In addition, right vlPFC activity was shown to be negatively correlated with social avoidance in SAD patients. In contrast, this correlation was reversed in healthy controls. Decreased vlPFC oxy-Hb suggests that SAD patients may have difficulty successfully recruiting emotion regulation-related brain regions. Liu et al. (2014) also adopted a verbal fluency task and found that MDD patients demonstrated smaller increases in oxy-Hb during task completion, and that bilateral PFC and antero-medial PFC oxy-Hb is correlated with depression severity. NIRS studies reporting observable differences in oxy-Hb between patient groups, as well as between patient groups and healthy individuals, demonstrate that NIRS is an effective imaging methodology in the study of the pathophysiology of patient disorders. Yet, given how research shows that state and trait emotion can have separate and combined effects on cognitive processing (Crocker et al., 2012), future studies need to consider the role of both state and trait emotion in their experimental design.

## Recommendations for future research

There are a number of aspects related to experimental design that NIRS researchers should consider when investigating the influence of emotion on cognition.

1. **Inclusion of sufficient experimental conditions**
  Altered neural processing has been observed in relation to both positive and negative stimuli (Glotzbach et al., 2011; Hoshi et al., 2011; Kreplin and Fairclough, 2013; Ozawa et al., 2014). Consequently, the inclusion of positive, negative, and neutral experimental conditions whenever possible will permit more accurate comparisons between studies and allow more precise conclusions about underlying neural substrates to be generated.
2. **Experimental task considerations**
  Task difficulty can influence PFC neural activity and mitigate the influence of emotion on task performance. Thus, the choice of experimental task needs to be carefully considered. Additionally, some studies do not attempt to relate neural activation to task performance and future work should include neural activation, emotion, and task performance in their analysis framework.
3. **Inclusion of baseline data and experimental conditions within analysis framework**
  It is also important for future work to report analysis for activation changes compared between conditions (experimental conditions and control conditions) as well as comparison of oxy-Hb activity in relation to baseline oxy-Hb activity. Reporting both may help to disentangle task-related changes in activation from emotion-related differences in activation (during a task). It would also be advisable for researchers to report the way in which their baseline recordings are taken as this is an important consideration when reflecting on the changes taking place in response to emotional processing.
4. **Inclusion of individual level and group level analysis**
  The content of emotional stimuli is subjective, and consequently PFC activation has been shown to vary across individuals in relation to emotional stimuli (Hoshi et al., 2011). We propose that researchers consider using individual level analysis to investigate these variations. This data would allow the investigation of any mitigating influence of emotion on task performance and underlying neural activity. Additionally, Tachtsidis and Scholkmann (2016) suggest that the strength of task-related systemic physiological change is dependent upon the emotional state of the individual, thus the inclusion of individual level analysis may be beneficial in avoiding false positives and false negatives in oxy-Hb analysis due to the influence of systemic physiological fluctuations.

## Conclusion

The growing field of emotion science stands to benefit from studies adopting NIRS to measure neural activation during emotional and cognitive tasks. NIRS is well suited to measuring PFC neural activity, and the PFC has been shown to be involved in the processing of emotional information. Many of the benefits that NIRS affords, including its portability, reduction in participant discomfort, and ability to study a wider range of behavioral tasks, make it an excellent tool for the study of neural functioning in healthy and patient populations. Conflicting data has been reported concerning emotion-related neural activation and it is hoped that the recommendations included in this article may help researchers to unravel some of these inconsistences in future research. Specifically, researchers need to adopt more consistent experimental designs, critically consider their choice of experimental task, and provide more detail regarding analysis to allow for effective comparison between studies and better explore the influence of emotion on cognition.

## Author contributions

RB: review concept, verification of analytic methods, main conclusions, article drafting. PE: verification of review concept, conclusion verification, proof reading. CT: verification of review concept, verification of analytic methods, conclusion verification, article drafting.

### Conflict of interest statement

The authors declare that the research was conducted in the absence of any commercial or financial relationships that could be construed as a potential conflict of interest.
